# When a Shadow Brings Peace: A Trauma-Related Visual Phenomenon in an Adolescent With Posttraumatic Stress Disorder

**DOI:** 10.7759/cureus.101987

**Published:** 2026-01-21

**Authors:** Ron Gabriel A Peji, Diane D Lipat, Alyssia M De Rojas

**Affiliations:** 1 Center for Psychotraumatology, Livence Mental Health, Silang, PHL; 2 Social Sciences Research Department, ADP and Co. Research Services and Consultancy, Silang, PHL

**Keywords:** adolescent trauma, art therapy, posttraumatic stress disorder, trauma-informed assessment, trauma-related hallucinations, visual perceptual experiences

## Abstract

Trauma-related perceptual experiences may resemble psychotic symptoms, creating diagnostic challenges in adolescent populations. We report the case of a Filipino preadolescent female diagnosed with posttraumatic stress disorder (PTSD) and major depressive disorder following prolonged intrafamilial sexual abuse. During trauma-focused psychotherapy incorporating expressive techniques, the patient consistently depicted and occasionally perceived a non-vivid, dark, cloud-like visual phenomenon that appeared exclusively during states of emotional calm, safety, or happiness. The experience was transient, purely visual, and associated with a subjective sense of peace. Importantly, the patient demonstrated intact insight, preserved reality testing, absence of distress, and no functional impairment related to the phenomenon. Comprehensive phenomenological assessment and differential diagnosis ruled out psychotic disorder, neurological causes, and mood-congruent psychosis. The visual experience was conceptualized as a non-psychotic, trauma-related perceptual phenomenon. This case highlights the importance of contextual, emotional, and insight-based assessment of perceptual experiences in traumatized adolescents to prevent misdiagnosis and inappropriate treatment.

## Introduction

Posttraumatic stress disorder (PTSD) is a trauma- and stressor-related disorder that may develop following exposure to actual or threatened death, serious injury, or sexual violence. It is characterized by the presence of intrusion symptoms (e.g., distressing memories or flashbacks), persistent avoidance of trauma-related stimuli, negative alterations in cognition and mood, and marked alterations in arousal and reactivity, with symptoms lasting for more than one month and causing clinically significant distress or functional impairment. In children and adolescents, PTSD may present with additional developmental features, including difficulties in emotional regulation, behavioral changes, and atypical perceptual experiences, particularly following chronic interpersonal trauma such as sexual abuse [1].

Major depressive disorder (MDD) is a mood disorder defined by the presence of at least one major depressive episode lasting a minimum of two weeks, characterized by persistent depressed mood or loss of interest or pleasure, accompanied by cognitive, emotional, and somatic symptoms such as feelings of worthlessness, impaired concentration, sleep disturbance, appetite changes, psychomotor changes, fatigue, and recurrent thoughts of death or suicide. In adolescents, MDD frequently co-occurs with PTSD, particularly in the context of prolonged trauma exposure, and may compound symptom severity, emotional numbing, and functional impairment [1].

While intrusive memories, hyperarousal, avoidance, and negative alterations in mood and cognition are well-recognized features of PTSD, trauma-related perceptual experiences that resemble hallucinations are increasingly reported in both adult and pediatric populations [2-5]. Trauma-related perceptual phenomena may include visual, auditory, or somatic experiences that are internally generated, transient, and context-dependent. Importantly, these experiences often occur with preserved reality testing and insight, distinguishing them from hallucinations associated with primary psychotic disorders [6-8].

Emerging literature suggests that hallucination-like experiences in PTSD differ phenomenologically from psychotic symptoms in terms of emotional valence, contextual triggers, insight, and functional impact [9,10]. Trauma and dissociation have been shown to shape perceptual experiences in ways that may appear psychosis-like while remaining anchored to trauma-related meaning and internal states [11]. In adolescents, the risk of misdiagnosis is heightened due to developmental factors, overlapping symptom presentations, and limited capacity to articulate subjective internal experiences [12,13]. Misattributing trauma-related perceptual phenomena to psychosis may result in inappropriate pharmacological intervention and failure to address the underlying trauma.

Trauma-focused psychotherapy refers to a group of evidence-based psychotherapeutic approaches that directly address traumatic experiences and their psychological consequences. These interventions aim to promote trauma processing, emotional regulation, and integration of traumatic memories within a safe therapeutic framework. Trauma-focused approaches commonly emphasize stabilization, gradual exposure to trauma-related material, meaning-making, and restoration of a sense of safety and control.

Expressive and art-based therapeutic approaches are frequently integrated within trauma-focused psychotherapy for children and adolescents. These modalities utilize nonverbal forms of expression - such as drawing, painting, or other creative activities - to facilitate the externalization of internal experiences that may be difficult to verbalize [14,15]. Art-based interventions can serve both therapeutic and diagnostic functions by providing insight into emotional states, symbolic representations of trauma, and perceptual experiences, while supporting emotional regulation and engagement in treatment. Broader integrative models have further highlighted links between trauma exposure and psychosis-spectrum presentations, underscoring the importance of nuanced differential diagnosis in trauma-exposed youth [16].

We present the case of an adolescent female with PTSD and comorbid major depressive disorder following prolonged intrafamilial sexual abuse, who reported a recurrent visual perceptual experience associated with emotional calm and preserved insight. This case highlights the clinical importance of differentiating trauma-related perceptual phenomena from psychotic features in adolescent practice and illustrates the value of expressive therapeutic approaches in understanding such experiences.

## Case presentation

The patient is a 12-year-old Filipino preadolescent girl who was referred by a school guidance counselor to the outpatient psychology service of a private mental health clinic for psychological evaluation and trauma-focused psychotherapy following the disclosure of prolonged intrafamilial sexual abuse. The patient presented specifically for psychological intervention, not for initial psychiatric consultation.

At the time of presentation, the patient was residing in a suburban community with her biological mother and stepfather. No other family members were living in the household. The patient is the only child. Her biological parents separated during early childhood, and she has had minimal contact with her biological father for several years. There was no reported involvement of grandparents or extended family members in her daily care.

The patient’s biological mother was employed in shift-based work outside the home, while the stepfather was unemployed during portions of the reported period of abuse, resulting in frequent unsupervised contact between the patient and the perpetrator. The sexual abuse was perpetrated by the stepfather and occurred repeatedly over an approximately one-year period, primarily during times when the mother was away for work. The mother reported that she was unaware of the abuse during its occurrence and became aware only after the patient’s disclosure. Following disclosure, the patient was immediately separated from the stepfather, and appropriate protective and safety measures were initiated. The patient was no longer residing with the perpetrator at the time of psychological intervention.

Medical, developmental, and family history

The patient had no history of significant medical illness, hospitalizations, neurological conditions, head injury, or chronic medical conditions. Her developmental milestones were reportedly achieved within expected age ranges, and there was no history of learning disability or neurodevelopmental disorder. No past psychiatric history or prior psychological treatment was reported before the traumatic exposure.

Family medical history was noncontributory. Family psychiatric history was notable for no known diagnoses of psychotic disorders, bipolar disorder, or major depressive disorder among first-degree relatives, as reported by the mother.

Premorbid functioning and psychosocial history

Premorbidly, the patient was described as socially engaged, emotionally expressive, academically functional, and compliant with authority figures. Teachers reported that she had been generally well-adjusted, with age-appropriate peer relationships and consistent school attendance prior to the onset of trauma.

Following the abuse, the patient demonstrated academic decline, social withdrawal, reduced classroom participation, and diminished interest in extracurricular activities. No history of substance use was reported.

Clinical presentation and symptom course

The patient reported that symptoms developed progressively during the one-year period of abuse and intensified following disclosure. Trauma-related symptoms included hypervigilance, avoidance of reminders associated with the perpetrator, emotional numbing, persistent fear, and physiological reactivity. Sleep disturbances were characterized by difficulty initiating sleep, frequent nighttime awakenings, trauma-related nightmares, and fear of sleeping alone.

Concurrent depressive symptoms included persistent low mood, anhedonia, social withdrawal, feelings of hopelessness, low self-worth, and reduced energy, resulting in functional impairment across home and school settings.

Assessment and diagnosis

The patient underwent a comprehensive clinical psychological assessment, consisting of multiple clinical interviews with the patient and her mother, behavioral observations, and symptom-based diagnostic evaluation guided by Diagnostic and Statistical Manual of Mental Disorders, 5th Edition (DSM-5-TR) criteria. Formal psychometric testing was deferred in the early phase of treatment due to emotional fragility and the need for stabilization.

The diagnosis of PTSD (DSM-5-TR: F43.10) was based on documented exposure to repeated sexual trauma, presence of intrusion symptoms (including trauma-related nightmares), avoidance behaviors, negative alterations in mood and cognition (including emotional numbing and self-blame), and hyperarousal symptoms persisting for more than one month and causing functional impairment. The diagnosis of major depressive disorder, severe (DSM-5-TR: F33.2) was supported by persistent depressed mood, marked anhedonia, social withdrawal, sleep disturbance, hopelessness, and functional decline.

Throughout assessment and treatment, the patient remained fully oriented, with preserved reality testing, coherent thought processes, and no evidence of delusions, formal thought disorder, or psychotic disorganization.

Medical and neurological evaluation

No radiological investigations, laboratory tests, or neurological imaging were conducted at the time of presentation, as there were no clinical indications suggesting neurological or medical etiology. There were no reported sensory deficits or neurological symptoms.

Psychiatric referral

Given the severity of depressive symptoms, the patient was referred for psychiatric evaluation. At the time of this report, no psychotropic medications had been initiated, and treatment proceeded with psychotherapy administered by a licensed clinical psychologist as the primary intervention, with ongoing monitoring for psychiatric referral as clinically indicated.

Therapeutic intervention

As presented in Table 1, the patient engaged in weekly trauma-focused psychotherapy sessions, each lasting approximately 50 minutes. The initial phase focused on safety, stabilization, psychoeducation, emotional regulation, and grounding techniques. As treatment progressed, cognitive-behavioral strategies were introduced to address trauma-related distortions such as shame and self-blame.

**Table 1:** Overview of the therapeutic goals and interventions across the course of treatment

Therapy Phase	Therapeutic Goals	Interventions	Session Frequency and Duration
Phase 1: Engagement and stabilization	Establish therapeutic alliance; ensure emotional safety; provide trauma-related psychoeducation; improve basic emotional regulation	Rapport-building; psychoeducation regarding trauma and PTSD symptoms; grounding techniques; basic affect identification	Weekly; 50 minutes per session
Phase 2: Symptom management and regulation	Reduce physiological arousal; improve affect tolerance; address sleep-related distress	Relaxation techniques; coping skills training; emotion regulation strategies; sleep hygiene education	Weekly; 50 minutes per session
Phase 3: Expressive and art-assisted processing	Facilitate nonverbal expression of internal experiences; support externalization of trauma-related emotions and perceptions	Art-assisted interventions including free drawing, symbolic imagery, self-figure drawings, color-based emotional representation, and guided drawing tasks	Weekly; 50 minutes per session
Phase 4: Trauma narrative development	Gradually process traumatic experiences; reduce avoidance; address maladaptive trauma-related cognitions	Narrative-based interventions; developmentally appropriate trauma narrative construction using verbal and expressive methods; cognitive restructuring targeting shame and self-blame	Weekly; 50 minutes per session
Phase 5: Interpersonal and family-oriented intervention	Strengthen caregiver support; promote emotional safety; reinforce protective boundaries	Joint sessions with biological mother; caregiver psychoeducation; boundary-setting discussions; communication enhancement	As clinically indicated; 50 minutes per session
Phase 6: Consolidation and relapse prevention	Reinforce adaptive coping strategies; enhance resilience; support functional recovery	Review and consolidation of skills; strengths-based interventions; relapse prevention planning	Weekly to biweekly; 50 minutes per session

Expressive and art-assisted therapeutic activities were incorporated, including (a) free drawing to represent emotional states, (b) symbolic self-figure drawings, (c) use of color and imagery to externalize internal sensations, and (d) narrative drawing exercises to support gradual trauma narrative construction. These activities facilitated nonverbal expression and emotional regulation while maintaining psychological safety.

Moreover, interpersonal and family-oriented interventions included (a) joint sessions with the biological mother to enhance emotional attunement, (b) psychoeducation regarding trauma responses, (c) boundary-setting discussions, and (d) reinforcement of safety and trust in caregiving relationships.

The patient was also referred for appropriate social support services to ensure continued protection and prevent further exposure to abuse.

Description of the visual phenomenon

During the course of trauma-focused psychotherapy, expressive techniques including art-assisted interventions were incorporated to facilitate emotional expression and trauma processing. Across multiple sessions, the patient consistently depicted a dark, cloud-like shadow positioned adjacent to her own figure in drawings that represented moments of emotional calm, safety, or happiness.

When gently explored in session, the patient reported that she occasionally perceived a similar shadow-like image in her environment outside of therapy. These visual experiences were described as transient, vague, and lacking detailed form, appearing as a dark cloud or shadow without distinct features, movement, or interaction. Notably, the phenomenon was reported to occur exclusively during periods in which the patient felt calm, emotionally regulated, or at peace.

The patient reported a subjective emotional response of peace when the shadow was present. She explicitly stated that she understood the image to be a product of her own mind and did not attribute it to an external entity or supernatural cause. There was no associated distress, fear, or behavioral response, and the experience did not interfere with her daily functioning. No auditory or other sensory components accompanied the visual perception. Throughout these reports, the patient demonstrated intact insight, orientation, and reality testing.

Differential diagnosis

Given the presence of a reported visual percept, a thorough differential diagnostic process was undertaken to evaluate whether the phenomenon represented a psychotic feature or an alternative trauma-related process.

Psychotic Disorder

Psychotic hallucinations were considered but deemed unlikely. The visual experience lacked persistence, vivid sensory detail, and multimodal involvement. Crucially, the patient demonstrated preserved insight, explicitly identifying the experience as internally generated. The phenomenon occurred exclusively in specific emotional states (calm or happiness), which is atypical of primary psychotic disorders. There were no accompanying delusions, thought disorganization, negative symptoms, or functional decline, further arguing against a psychotic disorder [17,18].

Trauma-Related Perceptual Disturbance

Trauma-related perceptual phenomena were considered the most plausible explanation. PTSD has been associated with transient, non-psychotic perceptual experiences, particularly in the context of dissociation or trauma-related symbolic processing. Such phenomena may differ phenomenologically from psychotic hallucinations in terms of insight, emotional context, and functional impact [2-5,9,10,19]. The emotional neutrality or positive affect associated with the image, as well as its symbolic consistency across expressive media, supported this interpretation.

Dissociative Phenomena

Dissociation-related imagery was also considered. The patient’s ability to externalize internal psychological states through imagery, coupled with preserved reality testing, is consistent with mild dissociative processes commonly observed in survivors of chronic interpersonal trauma [6-8,11,20].

Neurological or Medical Etiology

Neurological causes of visual disturbances were considered clinically unlikely due to the absence of neurological symptoms, lack of sensory complexity, emotional specificity of the experience, and stable cognitive functioning.

Based on the above considerations, the visual phenomenon was conceptualized as a non-psychotic, trauma-related perceptual experience rather than a manifestation of psychosis [2-5,19].

Clinical course and follow-up

The patient engaged in weekly trauma-focused psychotherapy over a total of 28 sessions, conducted across approximately seven months. Treatment was concluded following mutual agreement between the patient, caregiver, and clinician, based on sustained symptom improvement and functional stabilization.

Over the course of therapy, the patient demonstrated progressive reduction in core PTSD symptoms, including decreased hypervigilance, reduced trauma-related avoidance, improved emotional regulation, and significant improvement in sleep quality, with marked reduction in trauma-related nightmares. Depressive symptoms likewise improved, with increased engagement in age-appropriate activities, improved mood stability, enhanced self-esteem, and restoration of social and academic functioning. Overall quality of life, as assessed clinically through patient and caregiver reports, showed substantial improvement by the final sessions.

Formal psychometric reassessment was not conducted at termination, as initial phases of treatment prioritized stabilization and trauma processing; symptom change was monitored through serial clinical interviews, behavioral observations, and caregiver reports.

At the time of the final therapy session, the previously reported visual shadow-like percept was no longer experienced as distressing and occurred infrequently. When present, it remained brief, nonintrusive, and was consistently recognized by the patient as internally generated, with preserved insight. The phenomenon did not impair functioning and did not evolve in complexity or emotional valence.

At the last follow-up, the patient was residing exclusively with her biological mother and was no longer living with the stepfather. Protective and safety measures had been implemented following disclosure, and no further contact with the perpetrator was reported. No additional psychological therapies or psychiatric medications were initiated during or after the course of trauma-focused intervention.

## Discussion

This case highlights a clinically important presentation of a trauma-related visual perceptual experience in an adolescent with PTSD that mimicked hallucinations but did not meet diagnostic criteria for psychosis. Psychotic disorders are characterized by the presence of hallucinations, delusions, disorganized thinking, grossly disorganized or catatonic behavior, and/or negative symptoms, typically accompanied by impaired reality testing and limited insight into the pathological nature of the experiences [18]. In contrast, PTSD-related perceptual phenomena may occur in the absence of formal thought disorder, delusional beliefs, or deterioration in functioning, and are often contextually linked to trauma-related emotional or dissociative states [2-5,9,10].

In this case, several features argued strongly against a psychotic process. The visual phenomenon was transient, emotionally specific, and occurred exclusively during states of calm or emotional safety rather than during periods of distress or arousal. Importantly, the patient consistently demonstrated intact reality testing, defined as the ability to distinguish internal experiences from external reality, and maintained insight by recognizing the image as internally generated rather than externally imposed. There was no evidence of delusional interpretation, behavioral disorganization, or conviction regarding the objective reality of the percept, which are hallmarks of psychotic hallucinations [17,18]. The preserved insight and absence of distress or functional impairment further differentiated this experience from psychosis.

Trauma-related perceptual experiences in PTSD have been increasingly recognized as distinct from psychotic hallucinations in both phenomenology and clinical significance. Such experiences are often temporally linked to emotional states, dissociation, or symbolic trauma processing, and may carry personal meaning rather than persecutory or bizarre content [6-8,11,19]. Unlike psychotic hallucinations, which typically persist across contexts and are experienced as intrusive and externally generated, trauma-related imagery may fluctuate in frequency, intensity, and emotional valence while remaining anchored to the individual’s internal psychological state.

The consistency of the visual image across expressive modalities in this case suggests a symbolic internal representation rather than a fragmented perceptual disturbance. Art-assisted therapeutic techniques appeared to facilitate the externalization and containment of internal trauma-related experiences in a manner that was developmentally appropriate and emotionally regulated. Such imagery may reflect adaptive mechanisms of trauma integration rather than psychopathology, particularly when accompanied by preserved insight and emotional stability [14,15].

From a clinical perspective, this case underscores the importance of comprehensive phenomenological assessment when evaluating perceptual experiences in trauma-exposed adolescents. Clinicians should carefully assess reality testing, degree of insight, emotional context, functional impact, and associated cognitive or behavioral features before attributing perceptual experiences to psychosis. Failure to do so may lead to misdiagnosis, unnecessary exposure to antipsychotic medication, and delayed implementation of trauma-focused interventions [9,10,16].

Furthermore, this case highlights the diagnostic and therapeutic value of expressive and art-based approaches in pediatric trauma care. Such modalities may provide access to internal experiences that are difficult to verbalize while simultaneously offering critical diagnostic information regarding the nature of perceptual phenomena. Recognizing trauma-related perceptual experiences as part of a broader trauma response rather than psychosis can guide appropriate treatment planning, promote recovery, and reduce stigma for affected adolescents.

## Conclusions

This case illustrates that visual perceptual experiences in preadolescents with posttraumatic stress disorder are not inherently indicative of psychosis. The presence of preserved insight, emotional specificity, absence of distress, and lack of functional impairment supported a trauma-related rather than psychotic conceptualization of the reported visual phenomenon. Careful phenomenological assessment, including evaluation of emotional context and reality testing, is essential to differentiate trauma-related perceptual disturbances from primary psychotic disorders. Recognition of such distinctions may help prevent misdiagnosis and unnecessary pharmacological intervention while ensuring that trauma-focused therapeutic approaches remain central to treatment.
